# Expression of Concern: Exploring the determinants of exclusive breastfeeding among infants under-six months in Ethiopia using multilevel analysis

**DOI:** 10.1371/journal.pone.0328086

**Published:** 2025-07-15

**Authors:** The PLOS One Editors

The *PLOS One* Editors issue an Expression of Concern to alert readers that this article [1] was identified as one of a series of submissions for which we have concerns about authorship and adherence to the journal’s first and second publication criteria. We regret that these issues were not addressed prior to the article’s publication.

In addition, this article [1] reports a similar study and results as an article [2] that was published later by the same authors, and subsequently retracted [3] due to similarity with this article [1].
