# Supplementary material for: Exploring the determinants of exclusive breastfeeding among infants under-six months in Ethiopia using multilevel analysis
Source: PLoS One. 2021 Jan 13;16(1):e0245034. doi: 10.1371/journal.pone.0245034 (PMC7806157; doi:10.1371/journal.pone.0245034)
Supplement: Attachment — Submitted filename: Response to Reviwers.docx [file pone.0245034.s003.docx]

Rebuttal letter	Date: October/20/2020

PONE-D-20-23410

**Exploring determinants of exclusive breast feeding among infants under- six months in Ethiopia using multilevel analysis**

**Erkihun Tadesse**

**PLOS ONE**

Dear all,

We would like to thanks for these constructive, building and improvable comments on this manuscript that would improve substance and content of the manuscript. We considered each comments and clarification questions of editors and reviewers on the manuscript thoroughly. Our point-by-point responses for each comment and questions are described in detailed on the following pages. Further, the details of changes were shown by track changes in the supplementary document attached.

| **Editor questions/comments** | | **Response** |
| --- | --- | --- |
| **Methodology**  In the ethics statement in the Methods section and online submission information, please ensure that you have specified  (1) whether consent was informed and  (2) What type you obtained (for instance, written or verbal, and if verbal, how it was documented and witnessed). If the need for consent was waived by the ethics committee, please include this information.  -If you are reporting a retrospective study of medical records or archived samples, please ensure that you have discussed whether all data were fully anonymized before you accessed them and/or whether the IRB or ethics committee waived the requirement for informed consent. | | Thank you Editor for your constructive comment. We used the secondary data i.e. from 2016 Ethiopia Demographic health survey data for analysis. We amended the main manuscript file as well as in the online submission by adding:  All data used in this study were publicly available and aggregated secondary data with not having any personal identity. The data was fully available in the full DHS website ([www.measuredhs.com](http://www.measuredhs.com/)). |
| **Reviewer 1**   1. Definition of EBF should be added except allow of oral re-hydration saline | | Thanks reviewer for your constructive comment. We have amended the definition of EBF in the main manuscript using the WHO recent definition;  The World Health Organization (WHO) defines exclusive breastfeeding (**EBF**) as when 'an infant receives only breast milk, no other liquids or solids are given not even water, with the exception of oral rehydration solution, or drops/syrups of vitamins, minerals or medicines' |
| Line 165 the tense should not be future tense (will be) | | Thanks reviewer for your comments. We have replaced will be by was substituted by was. Since the aggregate value for all generated variables not normally distributed, it was categorized into groups based on the national median values and based on previous related studies. |
| 3. Line 178 categorizing poverty is not enough; you need to mentioned how you have calculated the wealth index with what were the variables name | | Thank you for your comments. Poverty categorization is done according to wealth index. Wealth is the value of all natural, physical and financial assets owned by a household, reduced by its liabilities. Household wealth is a measure commonly used in food security assessments. It gives an idea of households’ ability to access food, the severity of food insecurity and gives information about the economic situation of the food insecure. It is used to differentiate between the poorer and the wealthier households in food security related indicators, such as food consumption, and thereby provides information on how to target the food insecure.  There are several ways in which wealth, economic status of households and living standards can be measured. Income, expenditure and consumption are three common measurements. However, there are challenges in collecting and measuring income and expenditure accurately. An alternative is to use data on asset ownership and housing characteristics and combine this information into a proxy indicator such as the wealth index, which is created using principal component analysis (PCA). Asset ownership gives an indication of the longer-term economic status of a household and is less dependent on short-term economic changes compared with other wealth or poverty measures.  The wealth index measures relative wealth and, unlike a poverty line, is not an absolute measure of poverty or wealth. When referring to the wealth of households based on the wealth index we can talk about poor*er* and wealthi*er* households but we cannot conclude who is absolutely poor and wealthy. The wealth index quintiles divide the whole population into five equally large groups, based on their wealth rank. For example, in an area where only 10% of households fall below the poverty line, 40% of households will still fall into the two poorest quintiles and therefore be classified as the poorest.  The wealth index is commonly used in reports and analysis based on datasets from Demographic and Health Surveys (DHS) and is used to rank households into quintiles. The value of using the wealth index is especially recognized in contexts where reliable income and expenditure data is absent. The research questions related to the wealth index vary according to the different interests of the surveys. In DHS, the wealth index is chosen because of the major impact that wealth status has on household level health. It allows the investigator to identify the impact of wealth status on the research outcomes. DHS separates all interviewed households into five wealth quintiles to compare the influence of wealth on various population, health and nutrition indicators. The wealth index is presented in the DHS Final Reports and survey datasets as a background characteristic.  **Community level of poverty** was generated by recoding wealth index categorized as: “<25% wealth index as high poverty”, 25%-50% wealth index as Moderate poverty level and “>50% wealth index as Low poverty level of the communities”. A woman is said to be; poorest, if she lives in the household with the first quintile (1^st^ 20%) from the score of wealth index, poor; if she lives in the second quintile (2^nd^ 20%), middle; if she lives in the household with the third quintile (3^rd^ 20%), rich; if she lives in the household with the fourth quintile (4^th^ 20%), richest; if she lives in the household with the fifth quintile (5^th^ 20%) from the score of wealth index. |
| 4. Multi level logistic regression should be three outcome variables need to be mentioned in your write up? | | Thank you reviewer for the comment. In multinomial logistic regression there are three outcome variables but in multi-level logistic regression the outcome variable is binary (i.e. only two variables having dichotomous nature). Thus we considered multilevel logistic regression since the outcome is EBF having dichotomous nature (i.e. Yes if the infant is exclusively breast feed or not if the infant is not exclusively breast feed). |
| 5. Birth weight: In survey measuring the birth weight is very uncommon. You should describe how you have collected birth weight. | | Thanks again for the comment.  The Ethiopia demographic health survey data actually put birth weight of infants in grams, and it was categorized according to WHO criteria as low birth weight, normal and Macrosomia. |
| 6. You should consider only 4 ANC that is the current indicator for global comparison | | Thanks reviewer for the comment. We have amended the document as: Receiving antenatal care at least four times increases the likelihood of receiving effective maternal health interventions during the antenatal period. This is one of the indicators in the Global Strategy for Women’s, Children’s and Adolescents’ Health (2016-2030) Monitoring Framework, and one of the tracer indicators of health services for the universal health coverage (SDG indicator 3.8.1) as well as an additional indicator in the Sustainable Development Goals framework. But using only the fourth ANC visit may not show either the women follow all 4 visits or not. |
| 7. Need to provide a multi-level logistic regression table otherwise description is not enough. | | Thanks again for the comment. The multi-level logistic regression table was presented in the main document at the end after references section. |
| **Reviewer #2** | | |
| **In the method part**,  1.The authors should explain how they handle missing data. | Thank you for the comment. To manage the missing data, complete case analysis (CCA) was used, only those cases with complete data on some set of variables.  Since the total missing number is very low i.e. tolerable (31(2.6%)) which is found from three factors/independent variables (child comorbidities=4, time of breast feeding initiation=24, ANC visit=3), the multilevel analysis by itself took the complete case analysis as default. And no missing in the outcome variable of interest. | |
| 2. Also they need to explain how they done wealth index or how the EDHS conducts wealth index. | Thank you for your comments.  Wealth is the value of all natural, physical and financial assets owned by a household, reduced by its liabilities. Household wealth is a measure commonly used in food security assessments. It gives an idea of households’ ability to access food, the severity of food insecurity and gives information about the economic situation of the food insecure. It is used to differentiate between the poorer and the wealthier households in food security related indicators, such as food consumption, and thereby provides information on how to target the food insecure.  There are several ways in which wealth, economic status of households and living standards can be measured. Income, expenditure and consumption are three common measurements. However, there are challenges in collecting and measuring income and expenditure accurately. An alternative is to use data on asset ownership and housing characteristics and combine this information into a proxy indicator such as the wealth index, which is created using principal component analysis (PCA). Asset ownership gives an indication of the longer-term economic status of a household and is less dependent on short-term economic changes compared with other wealth or poverty measures.  The wealth index measures relative wealth and, unlike a poverty line, is not an absolute measure of poverty or wealth. When referring to the wealth of households based on the wealth index we can talk about poor*er* and wealthi*er* households but we cannot conclude who is absolutely poor and wealthy. The wealth index quintiles divide the whole population into five equally large groups, based on their wealth rank. For example, in an area where only 10% of households fall below the poverty line, 40% of households will still fall into the two poorest quintiles and therefore be classified as the poorest.  The wealth index is commonly used in reports and analysis based on datasets from Demographic and Health Surveys (DHS) and is used to rank households into quintiles. The value of using the wealth index is especially recognized in contexts where reliable income and expenditure data is absent. The research questions related to the wealth index vary according to the different interests of the surveys. In DHS, the wealth index is chosen because of the major impact that wealth status has on household level health. It allows the investigator to identify the impact of wealth status on the research outcomes.  DHS separates all interviewed households into five wealth quintiles to compare the influence of wealth on various population, health and nutrition indicators. The wealth index is presented in the DHS Final Reports and survey datasets as a background characteristic. | |
| 3-Besides, they need to explain community level factors in detail rather than simply describing in percentage. | Thanks reviewer for the comment.  **Community level variables** were generated by aggregating the individual characteristics in a cluster since EDHS did not collect data that can directly describe the characteristics of the clusters except place of residence. The aggregates were computed using the proportion of a given variables’ subcategory. Since the aggregate value for all generated variables not normally distributed, it will be categorized into groups based on the national median values and based on previous related studies.   - **Residence** is the only community level variable neither computed nor relabeled. - **Contextual region**: Ethiopia is demarcated for administrative purpose into 11 regions, which are classified in to agrarian, pastoralist and city based according to the living status of the population. The regions Tigray, Amhara, Oromia, Southern Nations Nationalities and peoples (SNNP), Gambella and Benshangul Gumuz were categorized as agrarian. The Somali and Afar regions were grouped to form pastoralist region and the Harari region, Addis Ababa and Dire Dawa city administrations were grouped to form city based populations. This contextual region was created by recoding of the 11 codes/regions of the country in to 3 categories according to the predefined context as described above. - The other community level variables were created by aggregating the individual characteristics. Using cluster variable (v001) the variables to be generated for community level variables were kept, reordered and collapsed. Then the collapsed variables produce totals and percentages that were used to create their categories based on previously similar studies. Finally labeling and recoding accordingly were done to merge with the individual level variables for final regression analysis. | |
| **Reviewer #3** | | |
| **Abstract** |  | |
| - When I started reading this manuscript, I had a feeling that this study about individual and community level determinants of EBF. I am sure other readers will have the same feeling by seeing the study title. However, in actual practice, authors considered many determinants including individual level factors, household level factors, community level factors and child level factors. | Thank you for your comments. Here we considered different level factors of EBF but all of them are bounded under individual level or community level for the sake of final analysis in two levels/clusters. The household level factors and child level factors were included under individual level factors of analysis. That is why we considered factors at individual level and community level. | |
| -Line 32, you abbreviated exclusive breastfeeding before in the first line, why do you write full here again? | Thanks for the comment. We described the word in short abbreviated form i.e. EBF and amended in the main manuscript. | |
| **Introduction**   lines 70-71 and 72-75 are totally different concepts, though they were written together without proper link.  - In lines 85 and 86 look these factors are only the factors of exclusive breastfeeding, though this is not right. | Thanks again for the comment. We have amended by presenting them in separate paragraph in the manuscript file.  These are not factors affecting EBF but we mentioned here some of the significant factors from previous litreatures. And amended in the main manuscript.  Even if the government of Ethiopia tried to promote and implement exclusive breast feeding practice for infants under six months of age but still there is poor improvement. This could be several contributing factors including Low level of educational status, maternal employment, operative delivery, late initiation of breast feeding, low antenatal and postnatal visits, increased production of formula foods and poor counseling of mother regarding EBF. Furthermore, Exclusive breast feeding was also influenced by community level factors including place of residence, contextual region, community level of maternal education, community level of ANC and PNC utilization, community level of employment, and community level of media exposure. | |
| **Methods**  -line 114, you analyzed EDHS data; you did not conduct this study. | Thank you for your comment. A study is conducted using primary or secondary data. In this research, we are using secondary data from the 2016 Ethiopia Demographic health survey data where data collected from the whole parts of the country i.e. nine regions and two city administrations. Thus, description of the study area where the data collected is one of the recommended protocols in research. That is why we described the study setting i.e. Ethiopia in the main manuscript. | |
| -Pease justification of selecting confounders. It seems you included all confounders without following any guidelines. I am also concerned about using the child comorbidity as confounders in this study. | Multivariable analysis is one of the methods of confounders controlling mechanism at analysis phase. At analysis phase, Bi-variable multilevel logistic analysis was computed and p-value up to 0.2 was selected to fit multi-variable multilevel logistic regression model. Those variables with p values less than or equal to 0.2 were taken for multivariable analysis to control confounding. Finally, in this study age of the infants, sex of the infants, infant comorbidities, wealth index, antenatal care visit, contextual region, community postnatal care visit and community level of employment status were statistically significant variables with exclusive breastfeeding in the final model using multivariable analysis by controlling confounding.  Child comorbidity is not a confounder rather it is truly associated with the outcome variable when the multivariable regression was performed. | |
| Results  Please revise ways of presenting your results in the text | Thanks for the comment. According to your query We made an exhaustive revision in presenting the result section. | |
| Discussion  -This section is very poor and authors failed to offer effective policy. | Thanks for the comment. According to your query we discussed our finding in detail. We also provided the strength and limitation of this study in detail. Furthermore the implication of the study was described clearly in detail at the end.  **Implications of the study**  Exclusive breastfeeding is one of the core indicators of infant and young child feeding, among strategies of reducing infant mortality and morbidity. It determines future growths and developments of the infants both in physical and mental. The study demonstrates that addressing individual and community level factors associated with exclusive breast feeding practice through policies and programs was essential to improve exclusive breastfeeding practice. Therefore, emphasis should be given on encouraging women to have ANC and PNC follow ups, where they may get information and education that will improve exclusive breastfeeding. The infants with comorbid conditions need attention since they have low exclusive breastfeeding tendency. Since maternal employment and wealth status have higher likelihood of exclusive breastfeeding, empowering women both economically and in their employment status is implicated. Therefore, interventions on individuals and community levels were demanded for saving lives of the infants and reduction of economic losses of a country. | |
