# Supplementary material for: Exploring the determinants of exclusive breastfeeding among infants under-six months in Ethiopia using multilevel analysis
Source: PLoS One. 2021 Jan 13;16(1):e0245034. doi: 10.1371/journal.pone.0245034 (PMC7806157; doi:10.1371/journal.pone.0245034)
Supplement: Attachment — Submitted filename: Reviewer comment.docx [file pone.0245034.s002.docx]

**Reviewer comment**

Generally, the paper is very smart, attractive and done based on scientific approach. Investigating this issue is at this time is so important and it’s really appreciated. However, here are few comments to be considered by the authors;

**In the method part**,

The authors should explain how they handle missing data.

Also they need to explain how they done wealth index or how the EDHS conducts wealth index.

Besides, they need to explain community level factors in detail rather than simply describing in percentage.

Otherwise, it is a nice work
